# Centric diatom diversity in the lower part of the Southern Bug river (Ukraine): the transitional zone at Mykolaiv city

**DOI:** 10.3897/phytokeys.178.64426

**Published:** 2021-05-20

**Authors:** Olena P. Bilous, Sergey I. Genkal, Jonas Zimmermann, Wolf-Henning Kusber, Regine Jahn

**Affiliations:** 1 Institute of Hydrobiology, NAS of Ukraine,12 Geroyiv Stalingrada Ave., Kiyv 04210, Ukraine; 2 I.D. Papanin Institute for Biology of Inland Waters of RAS, Settle Borok, Nekouz District, Yaroslavl Region 152742, Russia; 3 Botanischer Garten und Botanisches Museum Berlin, Freie Universität Berlin, Königin-Luise Str. 6–8, Berlin 14195, Germany

**Keywords:** Centric diatoms, diversity, Dnipro-Bug Estuary, ecology, phytobenthos, Southern Bug River, transitional zone

## Abstract

The diversity of centric diatoms is documented for the transitional zone of the lower part of the Southern Bug River (Ukraine) just before entering the Dnipro-Bug Estuary and compared to earlier results from the upstream sampling sites of the same river system. Benthic samples of the following sites were investigated: north of Mykolaiv City (approximately 5 km), in Mykolaiv City (near Varvarivskyi Bridge), and 5 km south of the city. Twenty-four centric diatom taxa belonging to 11 genera were identified, analysed, and documented by scanning electron microscopy (SEM) and light microscopy (LM). Among them, *Aulacoseira nivalis* is the first report for Ukraine, *A. islandica* and is the first confirmed record for the studied area since the 1930s. The maximum number of centric diatom taxa found in one station was 21, the minimum 10. *Melosira subglobosa* was the most common (documented in 57–80% of sites with centric diatoms) and abundant species 7.3–15.7% in relative abundance to all diatom taxa. The discovered diversity of taxa and its comparison with previous results is discussed with regard to the relevance of estuary zones in the research of diatoms.

## Introduction

Transitional waters are the continuum between freshwaters and coastal marine waters which according to the EU Water Framework Directive (The Directive 2000/60/EC) are defined as “bodies of surface water in the vicinity of river mouths which are partially saline in character as a result of their proximity to coastal waters but which are substantially influenced by freshwater flows”. All over the world, such waters attract scientific attention owing to the tremendous biodiversity that they sustain. The salinity is a major, if not the most important natural factor structuring the algal communities and explaining their variability within these ecosystems (Cebrián and Valiela 1999; Bode et al. 2005; Muylaert et al. 2006, 2009; Gameiro and Brotas 2010; Garmendia et al. 2011; Hartnett et al. 2011; Seoane et al. 2011). Furthermore, transitional waters have intrinsically higher productivity in comparison to open oceanic waters (Basset et al. 2013; Facca 2020). This may be attributed to the fact that such zones are deemed to be naturally stressed systems as they work as basins for runoff from their catchments and impact of saline waters from the sea (Zaldivar et al. 2008).

With shifts in the ecological ranges of organisms apparent in response to changes in freshwater flow, the ecological model of a transitional zone was studied in the Southern Bug River. A continuum of assemblages exists along the salinity gradient from the freshwater part of the River to the Dnipro-Bug estuary and within the estuary and the Black Sea. The Dnipro-Bug estuary consists of two parts: the wide Dnipro estuary (55 km long, up to 17 km wide), and the narrower Bug estuary (47 km long, from 5 to 11 km wide) with average depths of 6–7 metres and a maximum of 12 metres (Marynytch 1993). The salinity gradient in the Bug estuary itself varies in a wide range of 0.3–9.5 g/dm^3^, and the mean salinity equals 3.6‰ (Mykolaiv regional state administration 2019). These waters move upstream to Mykolaiv City forming a buffer zone, which may be defined as having two overlapping gradients formed by major saline stressors: freshwater species from the river and marine species from the estuary. Therefore, this area could be termed a transitional zone because it represents a transition community consisting of freshwater and marine species being at the edge of their ecological range.

In addition, the studied sites could also be impacted by severe stress from anthropogenic pressures. Precisely for this reason transitional waters are considered to be among the most impacted and ‘at risk’ ecosystems. Considering that, it is difficult to exclude these impacts on the species diversity of spatial and ecological gradual boundaries between these systems; nevertheless, salinity is considered as a prevailing stressor (van der Maarel 1990; Attrill and Rundle 2002).

The concept of this paper was inspired by the lack of knowledge in algal ecological variability, fluctuations and changes within transition zones, as well as by the importance of improving our understanding on the variability of different spatio-temporal scales and biological interactions (Smayda 1998). Of immense interest are the diatoms (Chromista, Bacillariophyta), which demonstrate a wide array of morphological, physiological, and behavioural traits and are a major component of marine and freshwater ecosystems (Kociolek et al. 2015). Many centric species are known from marine waters, but the group is present, with a considerably lower diversity, in freshwater habitats as well (Harwood and Nikolaev 1995). Based on available evidence, it appears that the majority of centric diatom genera occurring in freshwaters are ultimately derived from multiple immigration events from the marine realm (e.g., Alverson et al. 2007, 2011). Additionally, the transition zones greatly constitute ecosystems for rare and potentially neophytic centric diatom species. Hence, the investigation of diversity in this group of algae concerning salinity is of high interest and pertinence.

The centric diatom flora near the Mykolaiv region is not particularly well explored, however, some studies have been done (Genkal and Bilous 2015; Bilous et al. 2019). Our previous investigations revealed that these sites at the Southern Bug River have a higher diversity of algae in comparison to the sites over the entire water body explored in former studies (Bilous et al. 2012; Bilous et al. 2014; Belous 2016). Thus, the highlighted facts served as an incentive for continuation of the work and a more detailed analysis in order to better understand the development of different biotic components in response to changed salinity conditions. The aim of this work was the refined evaluation of the centric diatom species composition and its diversity in the transitional zone of the Southern Bug River. The results in this manuscript as well as the follow-up paper with a detailed pennate diatom species list could set the baseline and serve as a contribution to the discussions of high biodiversity in transitional zones.

## Materials and methods

**Figure 1.**
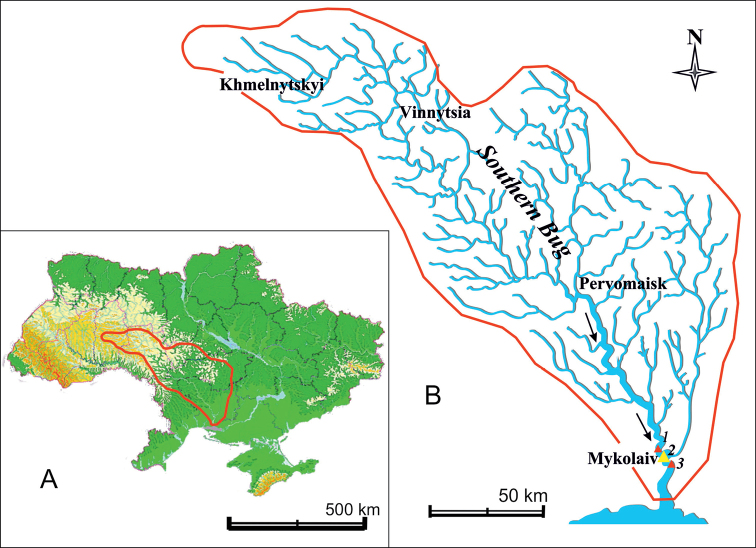
**A** Outline of Ukraine with the catchment area of the Southern Bug River in red **B** schematic drawing of the Southern Bug River with the sampling stations of the research in autumn 2017; the sampling site 2 – Mykolaiv City – was sampled also in the year 2013.

Benthic samples were collected in autumn 2017. The analysis of the species was divided into two parts: centric diatoms for this study and pennate diatoms for a follow-up study. The investigation was carried out at three sites assumed to have saline and freshwater impact on the lower portion of the Southern Bug River bed (Fig. 1): north of the Mykolaiv City (approximately 5 km) – 47°03'05"N, 31°52'35"E, in Mykolaiv City (near Varvarivskyi Bridge) – 46°59'07"N, 31°57'40"E, and 5 km south of the city – 46°48'59"N, 31°57'02"E. For the full picture of the studied area, our previous results (list of species at the Mykolaiv city site) from the sampling research in 2013, but with a different focus, were also considered.

Samples were taken by scraping diatoms from stones with a brush at a depth of 10–30 cm, homogenized and fixed with 70% ethanol. For LM and SEM investigations samples were cleaned from organic matter by standard procedures involving treatment with concentrated hydrogen peroxide and washing with deionized water. We focussed on benthic samples since they contain all diatoms, which occurred in the river at some time either as true benthos, as settled plankton or in between. Benthic samples treated by standard methods provide the best comparison with current and historic diatom studies.

Permanent diatom preparations were done by drying the cleaned samples on cover slips and mounting them with Naphrax (Kelly et al. 1998; European Committee for Standarization 2003, 2004; CEN 2014b). Light microscopic (LM) observations were performed by means of Axio.Imager2 (Carl Zeiss, Germany) equipped with oil immersion objective (×1000, DIC). Valve ultrastructure was examined on cleaned unsputtered material with a field scanning electron microscope FE-SEM (Hitachi SU8010) with 1.0 kV and 7.8–8.2 mm distance. LM slides and SEM stubs were studied at and are stored in the Algae Herbarium (B) Botanischer Garten und Botanisches Museum Berlin, Freie Universität Berlin.

Diatom identification was based on the books by Lange-Bertalot et al. (2017), Houk et al. (2010, 2014, 2017), Krammer and Lange-Bertalot (2000), Witkowski et al. (2000), and some selected works by Tanimura et al. (2004) and Aké-Castillo et al. (2012). Nine slides were investigated, at least 400 valves on each slide were counted following European Standard EN 14407 (CEN 2004b). The measurements of the taxa were made for at least 5 valves; most of the taxa were measured for 10–30 valves. In this paper, the focus is set on the centric diatoms, which include all diatoms from the modern systematic groups *Coscinodiscophyceae* and *Mediophyceae* (Medlin and Kaczmarska 2004).

## Results

The species diversity of centric diatoms from the three sites of the Southern Bug River bed (see Fig. 1) accounted for 24 taxa constituted by the following 11 genera: *Aulacoseira* Thwaites, *Actinocyclus* Ehrenb., *Conticribra* Stachura-Suchoples & D.M. Williams, *Cyclostephanos* Round, *Cyclotella* (Kütz.) Bréb., *Melosira* C. Agardh, *Minidiscus* Hasle, *Pleurosira* (Menegh.) Trevisan, *Skeletonema* Greville, *Stephanodiscus* Ehrenb., *Thalassiosira* Cleve.

The following 15 species were found in our previous investigation for this region of the river in 2013: *Aulacoseira subarctica* (O. Müll.) E.Y. Haworth, *Conticribra weissflogii* (Grunow) Stachura-Suchoples & D.M. Williams, *Cyclostephanos dubius* (Hust.) Round, *Cyclostephanos invisitatus* (M.H. Hohn & Hellerman) E.C. Theriot, Stoermer & Håk., *Cyclotella atomus* Hust., *Cyclotella choctawhatcheeana* A.K.S. Prasad, *Cyclotella meduanae* H. Germ., *Cyclotella meneghiniana* Kütz., *Melosira subglobosa* (Grunow) Houk, Klee & Tanaka, *Melosira varians* C. Agardh, *Skeletonema subsalsum* (Cleve-Euler) Bethge, *Stephanodiscus hantzschii* Grunow, *Stephanodiscus minutulus* (Kütz.) Cleve & J.D. Möller, *Thalassiosira incerta* I.V. Makarova and *Thalassiosira faurii* (Gasse) Hasle (Genkal and Bilous 2015). The survey in 2017 revealed 9 more centric diatoms for the investigated stations as specified in detail below (Table 1). However, the absence of *Melosira varians* in comparison to the previous investigation has to be noted. In summary, for the explored transitional zone the taxa belonged to the following systematic groups: class *Coscinodiscophyceae* (6 taxa) and class *Mediophyceae* (18 taxa), as orders these are *Melosirales* (2 taxa), *Aulacoseirales* (3 taxa), *Coscinodiscales* (1 taxon), *Triceratiales* (1 taxon) and *Thalassiosirales* (17 taxa), and as families *Melosiraceae* (2 taxa), *Aulacoseiraceae* (3 taxa), *Triceratiaceae* (1 taxon), *Hemidiscaceae* (1 taxon), *Thalassiosiraceae* (4 taxa), *Skeletonemataceae* (1 taxa) and *Stephanodiscaceae* (12 taxa).

**Table 1.** Species composition of centric diatoms at the investigated sites near Mykolaiv city in the Southern Bug River in the year 2017 (2013 added for station 2).

List of species	Stations			
	1: Upstream Mykolaiv	2: Mykolaiv (2017)	2: Mykolaiv (2013)	3: Downstream Mykolaiv
*Actinocyclus normanii*	+	+		+
*Aulacoseira islandica*	+	+		+
*Aulacoseira nivalis*	+	+		+
*Aulacoseira subarctica*		+	+	
*Conticribra weissflogii*		+	+	
*Cyclostephanos dubius*	+	+	+	
*Cyclostephanos invisitatus*		+	+	+
Cyclotella atomus var. atomus	+	+	+	+
Cyclotella atomus var. gracilis	+	+		+
*Cyclotella choctawhatcheeana*		+	+	+
*Cyclotella cryptica*				+
*Cyclotella marina*	+	+		+
*Cyclotella meduanae*		+	+	+
*Cyclotella meneghiniana*	+	+	+	+
*Melosira subglobosa*		+	+*	
*Melosira varians*			+	
*Minidiscus proschkinae*		+		+
*Pleurosira laevis*		+		+
*Skeletonema subsalsum*		+	+	
*Stephanodiscus hantzschii*	+	+	+	+
*Stephanodiscus makarovae*				+
*Stephanodiscus minutulus*		+	+	+
*Thalassiosira faurii*		+	+	
*Thalassiosira incerta*	+	+	+	
Taxa (number)	10	21	15	16

* in the publication of sampling research from 2013 (Genkal and Bilous 2015), this species was identified as *Melosira nummuloides* C. Agardh, however, Houk et al. (2017) provided evidence that the correct species name should be *Melosira subglobosa*.

With regard to the relative abundancies of the above counted taxa, *Melosira subglobosa* was the most abundant centric diatom with 7.3% – 15.7% relative abundance of all diatoms found in the benthic samples in 2017. Within centric diatoms only, its relative abundance varied from 57.1% to 80.4% and had its maximum values at the station 2 (80.4%) and 3 (80.3%). In contrast, *Actinocyclus normanii* had a relative abundance of 18.2% in station 3 (7.1% for station 2) and Cyclotella atomus var. atomus prevailed in station 1 with 14.3%. The other centric taxa made a much lower contribution to the diatom composition having less than 8% abundancies of all centric diatoms at each station.

### List of taxa with comments on their nomenclature, taxonomy, ecology and worldwide distribution

**Class Coscinodiscophyceae**

#### Order Melosirales Glezer in Glezer, Moiss. and I.V. Makarova 1990

**Family Melosiraceae Kütz. emend. Round, R.M. Crawford & Mann, 1990**

##### Genus *Melosira* C. Agardh, 1824

###### Melosira subglobosa

Taxon classificationPlantaeMelosiralesMelosiraceae

(Grunow) Houk, Klee & Tanaka in Fottea 17 (Supplement):17, pl. 17, figs 1–10; Pl. 19, figs 1–15; pl. 19, figs 1–11. 2017.

BC68D1C9-F040-5D34-8E5F-1E436D20D327

####### Synonyms.

Melosira borreri var. subglobosa Grunow, Melosira moniliformis var. subglobosa (Grunow) Hustedt.

####### Morphological description.

**Figure 2.**
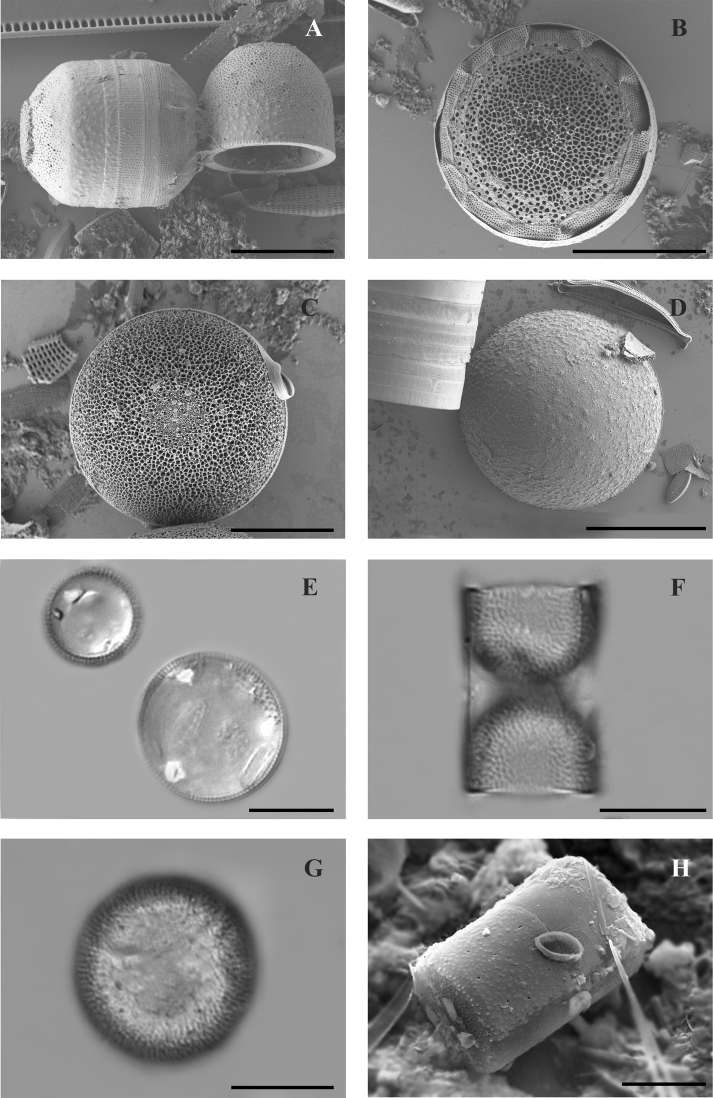
SEM (**A–D, H**) and LM (**E–G**) images of species found in the transitional zone at Mykolaiv city: **A–G***Melosira subglobosa* (**A, F** girdle views, **B–E, G**, valve views) **H***Melosira varians*, partial valve view and girdle view of complete frustule. Scale bars: 10 μm (**A–C, E–H**); 20 μm (**D**).

Frustule shape is cylindrical to approximately octagonal (Fig. 2A, F). Valve 12.5–26 μm in diameter, mantle height 3.1–8.5 μm. Valve face nearly octagonal with flat tops (Fig. 2B–E, G). Girdle with puncta forming straight and transverse rows 32–56 in 10 μm.

####### Ecology.

Marine and brackish, benthic-planktonic (tychoplanktonic) species, halophilic, widely distributed. Indicates significant organic pollution (eutrophication), α-mesosaprobiont (Kolpacov et al. 2014). This species prefers water enriched with dissolved organic matter, and is capable to switch from autotrophic to heterotrophic or mixed type of nutrition (Andreeva et al. 2008). Additionally, these diatoms are ubiquitous, and widely distributed in seas and estuaries of temperate zones in habitats with a moderate level with increased level of human impact (Kuzminova and Rudneva 2005; Ryabushko 2009).

####### Distribution.

*Melosira subglobosa* is one of the most common species at the sampling sites near the Mykolaiv area in the Southern Bug River (Table 1). For Ukrainian territory, it is known from the coastal zone in the northwestern part of the Black Sea, Crimean seaboard, and some estuaries.

For a worldwide distribution, it was recorded from coastal zones of Europe, and Asia; specifically, from Lake Aral, Baltic, Bering, Black, Caspian, Mediterranean, North and Norway Seas (Tsarenko et al. 2009).

###### Melosira varians

Taxon classificationPlantaeMelosiralesMelosiraceae

C. Agardh, Syst. Alg.: 64. 1816.

CD1BB2E3-04B2-5DF3-9ECF-47CA39D59AC4

####### Synonym.

*Aulacoseira varians* (C. Agardh) Simonsen.

####### Morphological description.

Frustule is cylindrical, valve flat, 15.7–46.6 μm in diameter with numerous small-scale granules, 5.7–15.5 μm high (Fig. 2H).

####### Ecology.

Common species for freshwaters in streams and lakes, as well as in slightly brackish waters, oligotrophic, eutrophic to dystrophic or polluted environments (Nardelli et al. 2016; Hofmann et al. 2018). Taxon has preferences of alkaline conditions (pH 7‒8.5), with moderate oxygen, regularly found in humid environments, requiring periodically high levels of nitrogen (Soltanpour-Gargari et al. 2011).

####### Distribution.

Valves were found near Mykolaiv city in the Southern Bug River in our previous sampling study (Table 1; Genkal and Bilous 2015). In Ukraine, it is known from the Southern Bug and lakes in the basins of the rivers Danube, Dnister, Siverskyi Donets, Desna, Prypiat, Dnipro and its reservoirs and estuary.

On the global level, it is a widely distributed taxon, known from Europe (i.e. Berlin, Germany, see Geissler and Kies 2003), Russia (Genkal et al. 2020), Asia (Iran), North America (Canada, USA), Africa (Egypt, RSA); Adriatic, Aral, Azov, Baltic, Barents, Black, Caspian, Kara, Mediterranean, North and Red Seas, and throughout North America (Stoermer and Julius 2003). It is also very common in Brazilian waters (Stoermer and Julius 2003; Tremarin et al. 2009; Nardelli et al. 2016).

#### Order Aulacoseirales V.A. Nikolajev ex Moiss. & I.V. Makarova, 1990

**Family Aulacoseiraceae Moiss., 1990**

##### Genus *Aulacoseira* Thwaites, 1848

###### Aulacoseira islandica

Taxon classificationPlantaeAulacoseiralesAulacoseiraceae

(O. Müll.) Simonsen, Bacillaria 2: 60, pl. 1, figs 1–10. 1979.

89E29735-8E80-5FAE-8A89-9FCEF001308E

####### Basionym.

*Melosira islandica* O. Müll., J. Wiss. Bot. 43 (1): 56, pl. 1, figs 3–6. 1906.

####### Synonyms.

Melosira islandica subsp. helvetica O. Müll., M. islandica subsp. vaenernsis A. Cleve.

####### Morphological description.

**Figure 3.**
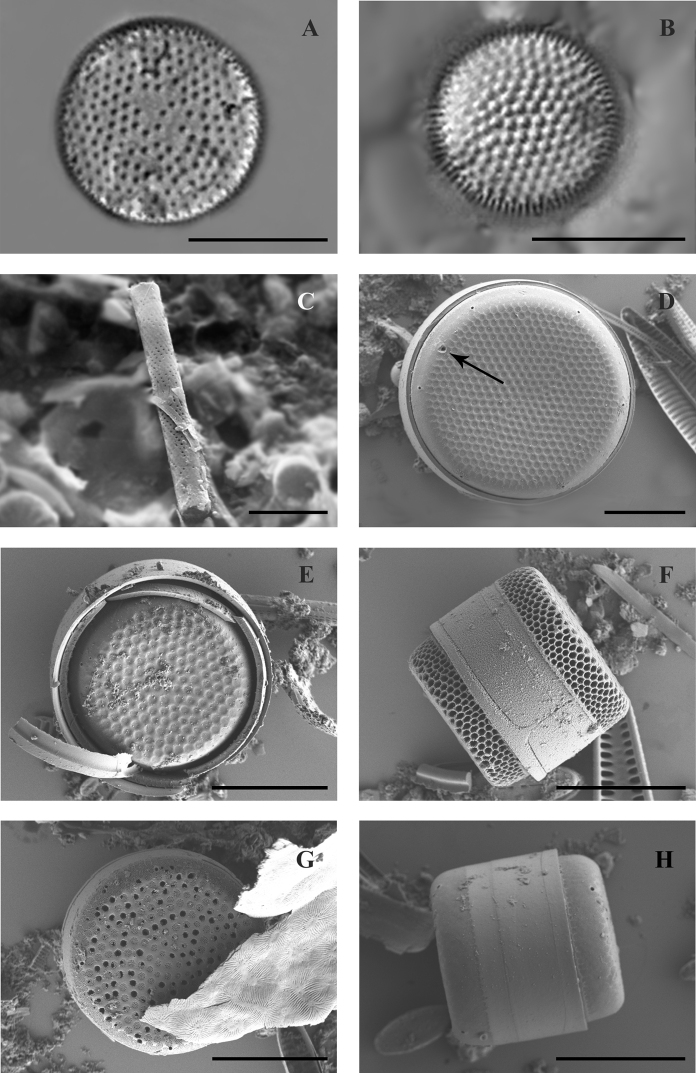
LM (**A,B**) and SEM (**C–H**) images of species found in the transitional zone at Mykolaiv city: **A***Aulacoseira islandica*, valve view **B***Aulacoseira nivalis*, valve view **C***Aulacoseira subarctica*, girdle view of complete frustule **D–H***Actinocyclus normanii* valve view (**D** pseudonodulus shown with arrow, **E, G**) and girdle view (**H, F**). Scale bars: 10 μm.

Frustule cylindrical, valve face flat with randomly located areolae, diameter is 13.2–14.4 μm (Fig. 3A). Curve of the valve with longitudinal areolae (12–14 in 10 μm) and transverse curly rows of areolae. The ringleiste is wide, connective spines are small-sized, sharp-ended, tear-drop-shaped or branched.

####### Ecology.

*Aulacoseira islandica* is most often occurring as planktonic or tychoplanktonic taxon in water bodies of different types in high latitude or high altitude oligotrophic to mesotrophic large waters (Houk et al. 2010; Stoermer and Julius 2013; Genkal et al. 2020). Sometimes this species may even cause a spring bloom (Stoermer and Julius 2013).

####### Distribution.

This species was observed in all sampling sites and is the first confirmed record for the whole basin of the Southern Bug River (Table 1). In the mid-1930s Swirenko (1941) found this species as Melosira islandica var. helvetica O. Müller in the lower part of the River starting from Mygea rapids and some other lower stations along the River bed to the mouth (Swirenko 1941). *Aulacoseira islandica* occurs in the Dnipro River basin and its reservoirs (Tsarenko et al. 2009).

It is also a common species for Europe (Bulgaria, Iran, Finland, Germany, Lithuania, Romania, Russia, Sweden, Ukraine), Asia (Georgia, Russia, Turkmenistan), North America (Canada, Greenland, USA); Barents, Bering and White Seas (Tsarenko et al. 2009).

###### Aulacoseira nivalis

Taxon classificationPlantaeAulacoseiralesAulacoseiraceae

(W. Sm.) J. English & Potapova, Proc. Acad. Nat. Sci. Philadelphia 158 (1): 39. 2009.

1ACFE8F8-99EB-591B-97F9-6551749199D7

####### Basionym.

*Melosira nivalis* W. Sm. Syn. Brit. Diat. 2, p. 58, pl. LIII, fig. 336. 1856.

####### Synonyms.

Melosira distans var. nivalis (W. Sm.) O. Kirchner, Aulacoseira distans var. nivalis (W. Sm.) E.Y. Haworth.

####### Morphological description.

Cells cylindrical, valve face flat, 7.5–12.8 μm in diameter, valve mantle 3.8–4.7 μm high. Valve face with rough areolae, located in intersecting diagonal rows (Fig. 3B).

####### Ecology.

This is a freshwater species, found growing in or near snow and in alpine pools (Houk et al. 2007; English and Potapova 2009) but was also reported for alkaline and acid waters (Kulikovskiy et al. 2016).

####### Distribution.

At the localities of upstream Mykolaiv, in Mykolaiv City and downstream Mykolaiv *Aulacoseira nivalis* is the first record for Ukraine (Table 1).

As for other countries, it is known from alpine and northern regions (Krammer and Lange-Bertalot 2000); frequently occurring in western North America (English and Potapova 2009) and as being abundant in lake sediments in Brazil.

###### Aulacoseira subarctica

Taxon classificationPlantaeAulacoseiralesAulacoseiraceae

(O. Müll.) Haworth in Round. Alg. aquat. environm.: 143–144. 1988.

07D5A744-8BC8-527F-8275-7BDF62DB5426

####### Basionym.

Melosira italica subsp. subarctica O. Müll., Jahrb. Wiss. Bot. 43: 78, pl. 2, figs 10–11. 1906.

####### Synonyms.

A. italica subsp. subarctica (O. Müll.) Simonsen.

####### Morphological description.

Frustules are usually low to high-cylindrical, valve face flat. Valve is 16.6 μm in diameter, 4.4 μm high, number of areolae lines on valve bend 15 in 10 μm, in the line 18 areolae in 10 μm. Ringleiste is wide, linking spines are large, sharpened (Fig. 3C).

####### Ecology.

*Aulacoseira subarctica* is a planktonic alga in lakes, rivers, reservoirs and temporary water bodies and is confined to higher latitudes. It usually appears in response to moderate increases in nutrients, supposedly to phosphorus concentrations controlling its presence and is disadvantaged by further enrichment (Chris et al. 2003).

####### Distribution.

Occurred in the Southern Bug River in Mykolaiv city (near Varvarivskyi Bridge) (Table 1). This species is known from the Dnipro River basin (Tsarenko et al. 2009).

*Aulacoseira subarctica* is widely distributed across Europe (Germany, Great Britain, Holland, Norway, Russia, Ukraine, Scandinavia), Asia (Russia, Japan, China), North America, Australia and New Zealand. The species is rare in the tropics, and positive identifications are lacking for Africa (Chris et al. 2003).

#### Order Coscinodiscales Round & R.M. Crawford in Round, Crawford and D.G. Mann 1990

**Family Hemidiscaceae Hendey ex G.R. Hasle, 1996**

##### Genus *Actinocyclus* Ehrenb., 1837

###### Actinocyclus normanii

Taxon classificationPlantaeCoscinodiscalesHemidiscaceae

(W. Greg.) Hust. in Abh. Naturwiss. Vereine Bremen 34 (3): 218, pl. 1, figs 5, 6. 1957.

195F3D4A-4BC6-5BCE-BA57-9BCA1C204527

####### Basionym.

*Coscinodiscus normanii* W. Greg. in Grev., Quart. J. Microsc. Sci. 7: 80, pl. 6, fig. 3. 1859.

####### Synonyms.

*Coscinodiscus curvatulus* Grunow, *C. fasciculatus* A.W.F. Schmidt, *C. normannicus* W. Greg., A. normanii f. subsalsus (Juhlin-Dannfelt) Hustedt

####### Morphological description.

The frustule is drum-shaped, the valve is flat or slightly concave or convex (Fig. 3D–H). Valve diameter 13.6–26.2 μm, height 3.4–5.2 μm. Areolae are arranged into sectors. The external openings of the process (4–6) are clearly visible on the curved outer surface the mantle (Fig. 3F, H). The pseudonodulus is located above the openings of the rimoportulae, it has a slight depression.

According to Hasle (1977), there are no significant taxonomic differences between A. normanii f. subsalsus and the nominate form (the ranges of valve diameters coincide), but there may be ecological preferences. Krammer and Lange-Bertalot (2000) did not identify forms, but gave so-called morphotypes that do not have a rank in nomenclature and, according to their data, in the *A. normanii* population from the Rhine region, a continuous range of forms was observed during the life cycle. Kozyrenko et al. (2008) synonymized A. normanii f. subsalsus with the nominate form and we adhere to their point of view.

####### Ecology.

Cosmopolitan, planktonic and phytobenthic, alkalibiontic and halophytic species, occurring in brackish inland waters influenced by anthropogenic nutrients and salts, waters with moderate to high conductivity (222–918 μS/cm), pH ranges from 7.8–8.6, at a water temperature between 8.0–25.7 °C and may serve as indicator of nutrient-rich habitats and polluted waters (Christie 2014; Vidaković et al. 2016).

####### Distribution.

*Actinocyclus normanii* is found sporadically in epilithic benthic samples from the the Southern Bug River at the three investigated stations (Table 1). It has previously been recorded for Ukrainian water bodies, especially for the Steppe zone noted in the monograph (Tsarenko et al. 2009) and for the Dnipro-Bug Estuary in particular (Vladimirova 1971; Zhukinskiy et al. 1989). It may travel upstream with highly mineralized waters from the estuary and appear near Mykolaiv City.

Upstream occurrences have been documented for Actinocyclus normanii f. subsalsus for Germany. According to diatom core analyses, this taxon reached the River Havel around 1900 (Schönfelder 1997). It was missing in Berlin (which is situated more than 200 km inland from the North and Baltic Seas) in the 1830s–1850s (Jahn and Kusber, unpubl. data from the Ehrenberg collection at BHUPM) but occurred in recent samples at the beginning of the 20^th^ century (Kolbe 1925; Geissler and Kies 2003) where it became an established part of the flora (Geissler and Kies 2003; Geissler et al. 2006). It was discussed by Schönfelder (1997) that a prerequisite for naturalisation might be the anthropogenically induced increase of salinity over the minimum value of salt tolerance. In other inland waters, e.g in the Czech Republic, the taxon occurred but did not establish (Fránková-Kozáková et al. 2007).

Additionally, it is a widely distributed species occurring in Europe, North and South America, the islands of the Atlantic Ocean, Africa, Asia, Australia, and New Zealand (Guiry and Guiry 2021). However, the species was considered invasive for Russia (see Kaštovský 2010; Korneva 2014) but was not included in the Handbook of alien species in Europe, outcome of the DAISIE (Delivering Alien Invasive Species Inventories for Europe) project (Handbook of alien species in Europe DAISIE 2009).

#### Class *Mediophyceae*

**Order Triceratiales Round & R.M. Crawford in Round, Crawford and Mann 1990**

**Family Triceratiaceae (F. Schütt) Lemmerm., 1899**

##### Genus *Pleurosira* (Meneghini) Trevisan, 1848

###### Pleurosira laevis

Taxon classificationPlantaeTriceratialesTriceratiaceae

(Ehrenb.) Compère in Bacillaria 5: 177, figs 1–17, 20, 39. 1982.

4A87A68D-2A7D-5446-B99C-F1FF014E0585

####### Basionym.

*Biddulphia laevis* Ehrenb. in Ber. Bekanntm. Verh. Königl. Preuss. Akad. Wiss. Berlin: 122. 1843.

####### Synonyms.

*Cerataulus laevis* Ralfs in A. Pritch., *C. polymorpha* Van Heurck, *Odontella polymorpha* Kütz.

####### Morphological description.

**Figure 4.**
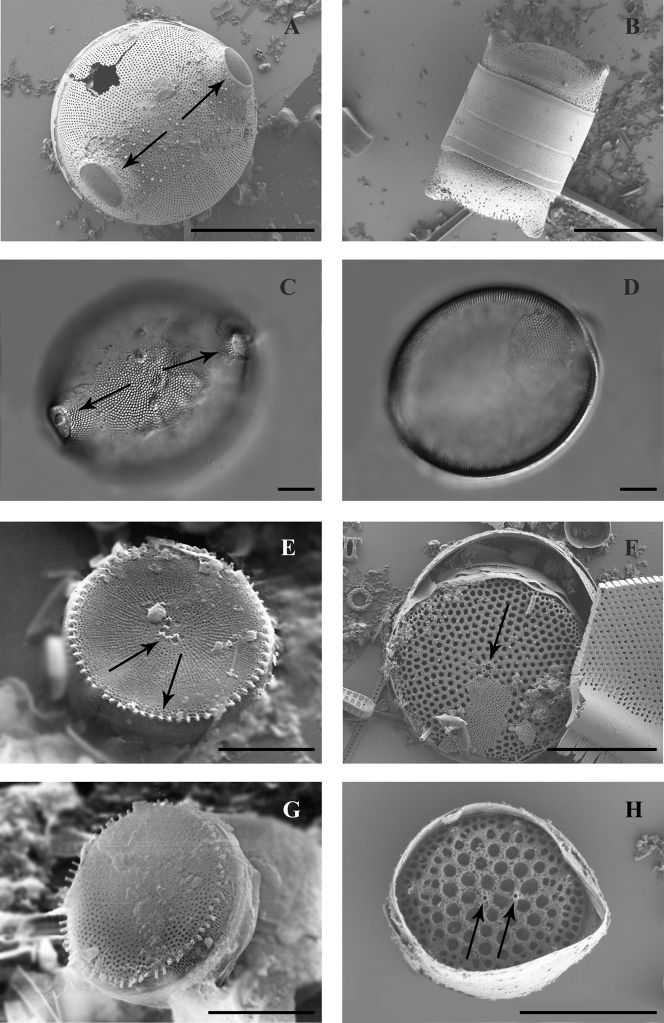
SEM (**A, B** and **E–H)** and LM (**C, D)** images of species found in the transitional zone at Mykolaiv city: **A–D***Pleurosira laevis***A, C** valve views with arrows shown the ocelli **B** girdle view **E***Conticribra weissflogii*, valve view with arrows shown central and marginal fultoportulae **F***Thalassiosira incerta*, valve view with arrow shown central fultoportulae **G***Thalassiosira faurii* , valve view **H***Minidiscus proschkinae*, valve view with arrow in the centre shown central fultoportula, and closer to the edge – rimoportula. Scale bars: 10 μm (**C–G**); 30 μm (**A**); 50 μm (**B**); 3 μm (**H**).

The frustule is cylindrical, valves are elliptical-rounded-oval, with diameter 39–61 μm, 15–17 areolae in 10 μm (Fig. 4A–D).

####### Ecology.

This taxon occurs in brackish and fresh water habitats, commonly found in estuaries of large rivers, also surviving in inland waters with high conductivity, this is a halophilic species. It has considerable abundance in epiphytic, benthic hard waters with relatively high electrical conductivity. Distributed and more abundant in warm-temperate and tropical waters, being mesohalobic, from alkalibiontic to alkaliphilic indicator, β-mesosaprobic, and eutraphentic (El-Awamri 2008).

####### Distribution.

Valves were found in benthic samples in the Mykolaiv city of the Southern Bug River and downriver (Table 1). For Ukrainian territory it was recorded for estuaries of the Black Sea, also reported for the Southern Bug River (Tsarenko et al. 2009).

*Pleurosira laevis* is quite cosmopolitan, distributed in the Boreal, near coasts of Europe (Czech Republic, Finland, Germany, Romania, Russia, Sweden, Ukraine), Asia (Korea, Turkey), South America (Brazil), Africa (Egypt), Hawaiian Islands; Azov, Black and Marmora Seas (Tsarenko et al. 2009; Park et al. 2017b). For Europe this taxon is considered an invasive species (Handbook of alien species in Europe DAISIE 2009). An upstream colonisation of *Pleurosira laevis* with help of different vectors for the River Labe was discussed by Fránková-Kozáková et al. (2007).

#### Order Thalassiosirales Glezer & I.V. Makarova, 1986

**Family Thalassiosiraceae M. Lebour, 1930**

##### Genus *Conticribra* Stachura-Suchoples & D.M. Williams, 2009

###### Conticribra weissflogii

Taxon classificationPlantaeThalassiosiralesThalassiosiraceae

(Grunow) Stachura-Suchoples & D.M. Williams, Eur. J. Phycol. 44: 482. 2009.

AC3606D4-CFAB-50B7-A11B-1C57677A76DB

####### Basionym.

*Micropodiscus weissflogii* Grunow in Van Heurck., 1885.

####### Synonyms.

*Eupodiscus weissflogii* Grunow, nom. inval., *Eupodiscus weissflogii* (Grunow) De Toni, *Thalassiosira weissflogii* (Grunow) G.A. Fryxell & Hasle

####### Morphological description.

The frustule has the form of a drum, valves are almost flat, diameter 24.4–26.6 μm, 8–10 marginal processes in 10 μm, 2–5 central processes (Fig. 4E).

####### Ecology.

*Conticribra weissflogii* is a planktonic diatom, from marine and brackish-water environments that also may occur in lacustric and riverine waters. It is reported to occur in a wide range of salinity 2–26‰ (representing oligohalobs to polyhalobs), especially at salinities above 5‰ (Stachura-Suchoples and Kulikovskiy 2014). This taxon tends to increase in population density with rising temperature (Lomas and Glibert 1999) as well as with eutrophication (Zheng et al. 2016). It is also known to grow in waters with relatively high pH, around 8–9.4 (Sala 1997).

####### Distribution.

This centric taxon appeared at the Mykolaiv site in the Southern Bug River; for Ukraine it was mentioned for the first time in our previous investigation (Table 1; Genkal and Bilous 2015), afterwards it was found in the tributaries of Dnipro in eastern and central parts of the country (Berezovskaya 2019; Kryvosheia and Kapustin 2019).

This is a widely distributed species: Europe, Asia, America (North and South), Africa, Australia and New Zealand; it was even found in Lake Baikal, also in the oceans over the world (Stachura-Suchoples and Kulikovskiy 2014; Genkal et al. 2020).

#### Order Thalassiosirales Glezer & I.V. Makarova, 1986

**Family Thalassiosiraceae M. Lebour, 1930**

##### Genus *Thalassiosira* Cleve, 1873

###### Thalassiosira incerta

Taxon classificationPlantaeThalassiosiralesThalassiosiraceae

I.V. Makarova in Bot. mater. otd. spor. rast. Bot. inst. AN SSSR 14: 50, pl. 1, figs 9–16. 1961.

253E3A75-3E56-591B-A103-B0980817E9A9

####### Synonym.

*Coscinodiscus bulla* M.H. Hohn & Hellerman.

####### Morphological description.

The frustule is cylindrical, valves approximately flat, 21.4–27.8 μm in diameter, in the middle of a valve 5 processes are located, 4–5 marginal fultoportulae in 10 μm, situated on the valve margin (Fig. 4F). The rimoportula is short-necked with an elongated, compressed narrow lip usually perpendicular to the margin.

####### Ecology.

The species was recorded as planktonic in water bodies of different types, typical for eutrophic/hypertrophic and highly saprobic marine, brackish, and fresh waters. It is euryhaline and eurythermal, in addition to being known as an alkaliphilic taxon (Okhapkin et al. 2016). *Thalassiosira incerta* was named an invasive taxon for Russia (Kaštovský 2010; Korneva 2014).

####### Distribution.

*Thalassiosira incerta* was recorded near Mykolaiv city in the Southern Bug River (Table 1). For Ukrainian territory, there are few records from reservoirs of the Dnipro River and some estuaries of the Black Sea and coastal waters near Crimea (Tsarenko et al. 2009).

This taxon is quite cosmopolitan, and distributed in the Boreal of Europe (Great Britain, Russia, Ukraine), Asia (Azerbaijan), North America (Canada, USA), Africa (Egypt); Aral, Black and Caspian Seas (Sims 1996; Kuo and Guo 2003; Okhapkin et al. 2016; Genkal et al. 2020).

###### Thalassiosira faurii

Taxon classificationPlantaeThalassiosiralesThalassiosiraceae

(Gasse) Hasle in Phycologia 17(3): 282, figs 61–65. 1978.

75AAF112-DFEB-55EA-A2C6-FED25FCCF265

####### Basionym.

*Coscinodiscus faurii* Gasse, 1975. PhD dis. Univ. Paris VI, Vol. II: 24, pl. 32, figs 1, 2. 1975.

####### Morphological description.

The frustule is low-cylindrical, valves are flat, 21.4–25.5 μm in diameter, central processes located on the valve ingroups, 6 marginal fultoportulae in 10 μm (Fig. 4G).

####### Ecology.

Planktonic and benthic in freshwater reservoirs, lakes, rivers, and fossil. Lack of information for this taxon must be noted, but it is known that this species is very sensitive to salinity (Roubeix et al. 2014).

####### Distribution.

*Thalassiosira faurii* (Gasse) Hasle occurred near Mykolaiv city in the Southern Bug River (Table 1). This taxon was registered for the Dnipro River and its reservoirs, mouth of the Danube in the Black Sea (Maystrova et al. 2007; Roubeix et al. 2014).

Worldwide distribution shows this species to occur in some European countries (Hungary, Russia), Asia (Russia), Africa (Ethiopia, Tanzania, Kenia, Kongo) (Tsarenko et al. 2009; Roubeix et al. 2014; Genkal et al. 2020).

#### Order Thalassiosirales Glezer & I.V. Makarova, 1986

**Family Thalassiosiraceae M. Lebour, 1930**

##### Genus *Minidiscus* Hasle, 1973

###### Minidiscus proschkinae

Taxon classificationPlantaeThalassiosiralesThalassiosiraceae

(I.V. Makarova) J.S. Park & J.H. Lee in J.S. Park et al., PLoS ONE 2(9): 18. 2018.

E8CC21D0-3F1A-5225-9F24-8FF5A667C776

####### Basionym.

*Thalassiosira proschkinae* I.V. Makarova in Makarova, Genkal and Kuzmin, Bot. Zhurn. 64(7): 922, pl. 1, figs 1–7. 1979.

####### Morphological description.

**Figure 5.**
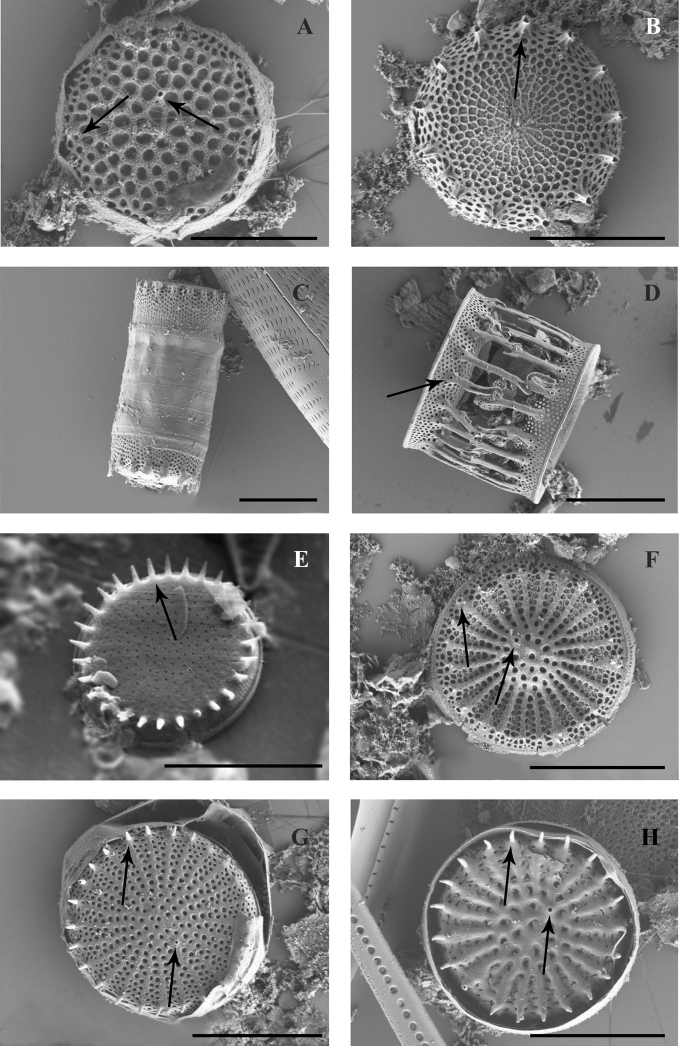
SEM images of species found in the transitional zone at Mykolaiv city (**A, B, E–H** valve views, **C, D** girdle views): **A***Minidiscus proschkinae* with arrows showing rimoportula and marginal fultoportula **B–D***Skeletonema subsalsum* with arrows showing marginal fultoportulae **E***Stephanodiscus hantzschii* with arrow shown spines **F***Stephanodiscus makarovae* with arrows showing marginal and central fultoportulae **G, H***Stephanodiscus minutulus* with arrows showing spines and central fultoportula. Scale bars: 3 μm (**A**); 4 μm (**B, F, H**); 5 μm (**C, D, G**); 10 μm (**E**).

The frustule is cylindrical, valve flat, diameter 3.8–4.9 μm, areolae polygonal, in quantities 25 in 10 μm, near centre of a valve, the central process and rimoportula are located (Figs 4H, 5A).

####### Ecology.

This planktonic taxon has been mainly found in estuaries characterized by low salinity and high turbulence, and in seas, but also in freshwaters and may be a halophile indicator (Makarova 1988; Park et al. 2017a; Barinova et al. 2019).

####### Distribution.

Valves were found in benthic samples in Mykolaiv city of the Southern Bug River and downriver (Table 1). For Ukrainian territory it was recorded for estuaries of the Black Sea and nearshore regions (Tsarenko et al. 2009; Genkal and Terenko 2014).

*Minidiscus proschkinae* is widely distributed across estuaries and seacoasts of Europe (Germany, Great Britain, Netherlands, Russia, Ukraine), Asia (Azerbaijan, China), Argentina; Azov Sea, Baltic Sea, Caspian Sea (Park et al. 2017a).

#### Order Thalassiosirales Glezer & I.V. Makarova, 1986

**Family Skeletonemataceae M. Lebour, 1930**

##### Genus *Skeletonema* Grev., 1865

###### Skeletonema subsalsum

Taxon classificationPlantaeThalassiosiralesSkeletonemataceae

(A. Cleve) Bethge in Ber. Deutsch. Bot. Ges. 46(5): 343, pl. 2, figs 1–11. 1928.

C11EEE85-5985-5D70-9267-28DEB02E1993

####### Basionym.

*Melosira subsalsa* A. Cleve in. Arch. Hydrobiol. 7: 509, fig. 1. 1912.

####### Morphological description.

The frustule is cylindrical, valves are flat or slightly convex, diameter 7.8–12.7 μm (Fig. 5B). Frustules are connected with marginal flat-spoon fultoportulae providing a very close connection between valve mantles (Fig. 5C, D).

####### Ecology.

Planktonic taxon, preferring low salinities, usually occurring in salinities up to 15‰, recorded mainly for the brackish waters, however, is known from rivers, lakes, inland seas, coastal waters, and marshes, and often associated with eutraphentic conditions, temperate taxon, alkaliphilic (Hasle and Evensen 1975; Sarno et al. 2005; Hofmann et al. 2018). In addition, in the DAISIE database *Skeletonema subsalsum* is considered to be an invasive species for Russia’s and Ireland’s water bodies (Kaštovský 2010; Korneva 2014) but is not listed in the *Handbook of alien species in Europe* (2009).

####### Distribution.

This taxon was found at the site of Mykolaiv city in the Southern Bug River (Table 1). *Skeletonema subsalsum* is widely distributed in Ukrainian waters, known from the Dnipro River in its freshwater reservoirs, in estuaries connected to the Black Sea and from coastal waters of different river basins as well as Crimea coast.

It is a cosmopolitan species, known from the Boreal, Europe (Finland, Germany, Ireland, Italy, the Netherlands, Romania, Russia, Sweden, Ukraine), North America (Canada, USA); Baltic, Black and Caspian Seas. (Gibson et al. 1993; Krammer and Lange-Bertalot 2000; Tsarenko et al. 2009; Genkal et al. 2020).

#### Order Thalassiosirales Glezer & I.V. Makarova, 1986

**Family Stephanodiscaceae I.V. Makarova in Glezer and Makarova 1986**

##### Genus *Stephanodiscus* Ehrenberg, 1845

###### Stephanodiscus hantzschii

Taxon classificationPlantaeThalassiosiralesStephanodiscaceae

Grunow in Cleve and Grunow in Bih. Kongl. Sven. Vet. Akad. handl. 17(2): 115, pl. 7, fig. 131. 1880.

D62072E2-0FAA-52A1-B0EA-8932B895C2DC

####### Synonyms.

*Stephanodiscus hantzschianus* Grunow, S. hantzschii var. delicatula A. Cleve, S. hantzschii var. zachariasii (Brun) Fricke, *S. zachariasii* Brun.

####### Concept synonym.

*Cyclotella operculata* sensu Hantzsch in Rabenhorst, Fl. Alg. Eur.: N 1104. 1861.

####### Morphological description.

The frustule is low-cylindrical, valves flat 13.6–21.4 μm in diameter, striae multiseriate with 6–7 in 10 μm, central processes are absent, spines large and pointy, growing from each rib (Fig. 5E).

####### Ecology.

Planktonic in lakes and rivers, indifferent, alkaliphilic, α-mesosaprobic, eutraphentic serving as an indicator of eutrophication in rivers, reservoirs, lakes worldwide mostly because of phosphorus loads (Håkansson and Stoermer 1984; Van Dam et al. 1994; Burge and Edlund 2016; Hofmann et al. 2018).

####### Distribution.

*Stephanodiscus hantzschii* was identified for the Southern Bug River in Mykolaiv city and downstream (Table 1). It is widespread taxon in Ukrainian water bodies: the rivers Danube, Dnister, Southern Bug, Siverskyi Donets, Dnipro and its reservoirs, coastal waters.

It is a cosmopolitan species, known from Europe (Belarus, Bulgaria, Finland, France, Germany, Moldova, Norway, Romania Russia, Ukraine), Asia (Armenia, China, Georgia, Japan, Korea, Mongolia, Russia, Tadjikistan, Uzbekistan), North America (Canada, USA); Aral, Azov, Baltic, Black and Caspian Seas (Krammer and Lange-Bertalot 2000; Tsarenko et al. 2009; Genkal et al. 2020).

###### Stephanodiscus makarovae

Taxon classificationPlantaeThalassiosiralesStephanodiscaceae

Genkal in Nov. Syst. Nizsh. Rast. 15: 13, pl. 2, fig. 1. 1978.

F7849DE9-42DC-53DA-ABB2-76945D6B1C0C

####### Morphological description.

Frustule disciform, valve with slightly convex or concave centre, frequently flat, diameter 5.9–8.3 μm, striae are double, rarely triple, in numbers of 14–16 in 10 μm. One central process is present. Spines pointy, small-scale, growing from each costa (Fig. 5F).

####### Ecology.

Planktonic in rivers, lakes and reservoirs, freshwater, but mainly in mesotrophic-eutrophic water bodies. In addition, reported occurrence of this species in high numbers in highly mineralized waters (Genkal 2007; Genkal et al. 2009; Tsarenko et al. 2009).

####### Distribution.

*Stephanodiscus makarovae* was observed downstream from Mykolaiv city in the Southern Bug River (Table 1). It was observed in the Dnipro River and its reservoirs, the Danube River and in the coastal area of the Black Sea (Genkal et al. 2009; Tsarenko et al. 2009; Genkal and Terenko 2014).

As for general distribution, this taxon has only a few records around the world – Europe (Russia, Ukraine), Asia (Armenia, Russia), Africa (Egypt) (Kulikovskiy et al. 2016; Genkal et al. 2020).

####### Comments.

According to Houk et al. (2014), *S. makarovae* (Genkal 2007) was erroneously included in the synonym *C. delicatus* (Genkal) Casper & Scheffler, since, according to the diagnosis, the marginal fultoportulae of *S. makarove* have 2 satellite pores, and for *C. delicatus* 3. The difference in the number of satellite pores at the marginal fultoportulae in centric diatoms is a good diagnostic feature. For *S. makarovae*, the valve relief also varies from flat to slightly convex or concave, and there are also other morphological differences (see same publication Genkal 2007). As for the transfer of *S. makarovae* to the genus *Cyclostephanos*, this is a debatable issue and molecular genetic studies are needed.

###### Stephanodiscus minutulus

Taxon classificationPlantaeThalassiosiralesStephanodiscaceae

(Kütz.) Cleve & J.D. Möller, Collect. Diat.: 300. 1882.

0444550A-6D09-55C9-BD0B-C0F684DADD0E

####### Basionym.

*Cyclotella minutula* Kütz. Kieselschal. Bacill.: 50, pl. 2, fig. 3. 1844.

####### Synonyms.

S. astraea var. minutula (Kütz.) Grunow, *S. minutulus* (Kütz.) Round, *S. parvus* Stoermer & Håk., *S. perforatus* Genkal & Kuzmin, S. rotula var. minutulus (Kütz.) R. Ross & P.A. Sims.

####### Morphological description.

**Figure 6.**
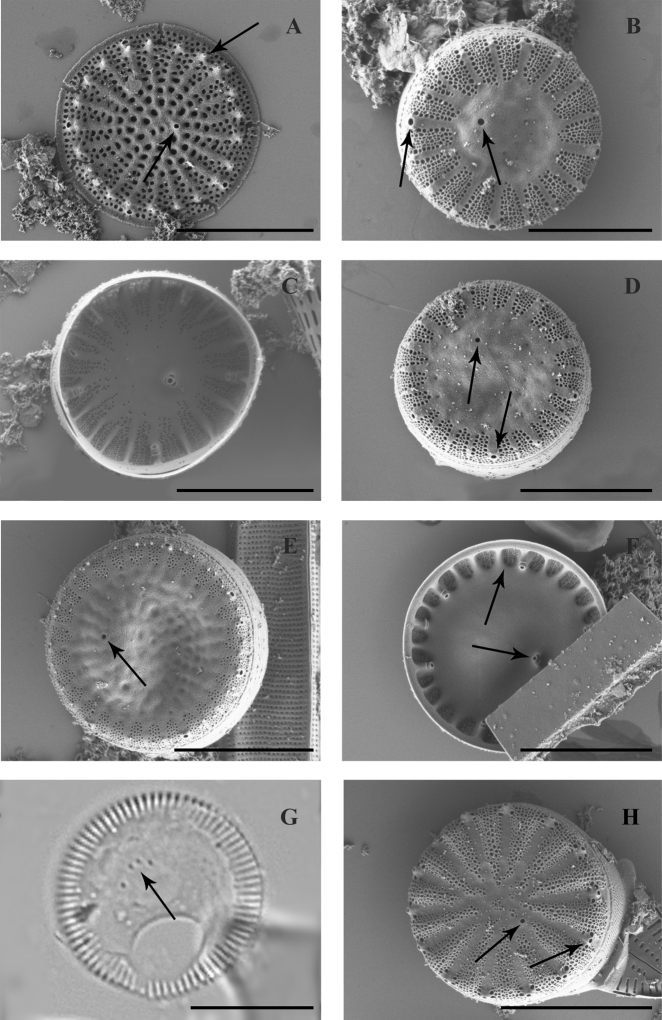
SEM (**A–F, H**) and LM images (**G**) of species found in the transitional zone at Mykolaiv city, valve views: **A***Stephanodiscus minutulus* with arrows shown marginal and central fultoportulae **B, C**Cyclotella atomus var. atomus with arrows shown marginal and central fultoportulae **D–F**Cyclotella atomus var. gracilis with arrows shown marginal and central fultoportulae on **D** picture, only central fultoportula on **E** and central fultoportula and alveolae on (**F**) **G***Cyclotella choctawhatcheeana* with arrow shown central fultoportulae **H***Cyclotella cryptica* with arrows shown marginal and central fultoportulae. Scale bars: 3 μm (**B, C**); 4 μm (**D, F**); 5 μm (**A, E, H**); 10 μm (**G**).

The frustule is disciform, valves flat or with slightly convex or concave centre, 8.8–9.1 μm in diameter, striae double to triple, numbering 10 in 10 μm (Fig. 5G, H). A central process is present. The spines are short, growing from each rib (Figs 5G, H, 6A).

####### Ecology.

It is a planktonic taxon, described as eutraphentic (Hofmann et al. 2018). *Stephanodiscus minutulus* reaches greatest abundance in productive nearshore regions, in the mouths of large rivers and coastal embayments. This taxon is an indicator of increased TP concentrations, alkaliphilic, mesosaprobic indicator (Stoermer and Yang 1969; Bradbury et al. 2002; Reavie and Kireta 2015).

####### Distribution.

*Stephanodiscus minutulus* occurred at the Mykolaiv city and downstream the Southern Bug river sites (Table 1). This taxon is distributed over the entire territory of Ukraine, common for such rivers as the Dnipro with its reservoirs and tributaries, the rivers Dnister, Danube, Siverskyi Donets, estuaries of main rivers (Tsarenko et al. 2009).

Concerning worldwide distribution, *Stephanodiscus minutulus* is a widespread taxon, recorded for Europe (i.e. Estonia, Finland, France, Germany, Great Britain, Hungary, Ireland, Moldova, Norway, Russia, Ukraine), Asia (Armenia, Georgia, Iran, Israel, Russia, Japan), North America (Canada, Greenland, USA) (Tsarenko et al. 2009; Kiss et al. 2012; Kulikovskiy et al. 2013; Genkal et al. 2020).

####### Comments.

Houk et al. (2014) considered *Stephanodiscus minutulus* to be different from *Stephanodiscus parvus* (and Stoermer and Håkansson 1984) and noted the main difference between *S. minutulus* and *S. parvus* the convex-concave valve relief in contrast to flat valves, respectively. However, many authors have shown that in *S. minutulus* the valve relief varies from convex-concave to flat, and therefore *S. parvus* was treated as a synonym (see Genkal 2010), and we adhere to this point of view.

#### Order Thalassiosirales Glezer & I.V. Makarova, 1986

**Family Stephanodiscaceae I.V. Makarova in Glezer and Makarova 1986**

##### Genus *Cyclotella* (Kütz.) Bréb., 1838

###### Cyclotella atomus Hust. var. atomus

Taxon classificationPlantaeThalassiosiralesStephanodiscaceae

in Arch. Hydrobiol. 15: 143, pl. 9, figs 1–4. 1938.

8A17A8A8-9F4C-5C1D-8660-45680D1A8083

####### Morphological description.

Frustule low-cylindrical, central part of the valve is slightly tangentially undulated, 3.6–5.6 μm in diameter, clear boundary between regional and central zones absent, 10–15 striae in 10 μm, and a central process (Fig. 6B, C).

####### Ecology.

Euplanktonic species, that may exist in marine, brackish or nearshore areas and freshwaters, indicating eutraphentic, α-mesosaprobous conditions and often associated with polluted, warm nutrient-rich water, however particularly tolerating high total phosphorus loads (Denys 1991; Van Dam et al. 1994; Yang et al. 2005; Lowe 2015), halophilic, alkaliphilic, tolerates higher ion concentrations and frequent osmotic stress as well as high temperature conditions and turbulence (Krammer and Lange-Bertalot 2000).

####### Distribution.

Valves were found at all investigated sites of the Southern Bug during this research (Table 1). For Ukrainian territory, it has been reported for the Dnipro River (Maystrova et al. 2007).

In general, *Cyclotella atomus* is a cosmopolitan species (Krammer and Lange-Bertalot 2000), widespread in freshwater and marine environments in North America, Europe, and Asia, and has also been recorded from Argentina and South Africa (Poulíčková 1993; Medioli and Brooks 2003; Tanimura et al. 2004; Yang et al. 2005; Wojtal and Kwandrans 2006; Genkal et al. 2020).

###### Cyclotella atomus var. gracilis

Taxon classificationPlantaeThalassiosiralesStephanodiscaceae

Genkal & K.T. Kiss in Hydrobiologia 269: 43, figs 10–16. 1993.

FC8618A4-C300-549F-A0FB-42B068B05478

####### Morphological description.

The frustule is low-cylindrical, central part of valve is slightly tangentially undulated, valves 4.6–7 μm in diameter, and a clear boundary between regional and central zones is present, 15–20 wedge-shaped striae in 10 μm, with central process (Fig. 6D, E).

####### Ecology.

Planktonic in rivers, lakes, freshwater, eutraphentic (Hofmann et al. 2018). It is regarded as an euryhaline species (Kiss et al. 2012).

####### Distribution.

Cyclotella atomus var. gracilis is here first reported for the studied area, and was found at all investigated sites during this study (Table 1). In turn, its existence was reported in Dnipro waters (Maystrova et al. 2007), as well as for the Danube River (Genkal and Ivanov 1990).

This species is cosmopolitan, i.a. it was recorded for European waters (Kiss et al. 2012; Genkal et al. 2020).

###### Cyclotella choctawhatcheeana

Taxon classificationPlantaeThalassiosiralesStephanodiscaceae

A.K.S. Prasad., 1990; emend. Genkal. Biol. vnutr. vod. 2: 1–10. 2012.

A1B2DA94-A46C-513B-AF59-405D68801B1A

####### Synonym.

*Cyclotella hakanssoniae* Wendker, Nova Hedwigia 52: 360. 1991.

####### Morphological description.

Frustule low-cylindrical, central part of valve tangentially undulated, valves 9.1–12.3 μm in diameter, 12–14 striae in 10 μm, 1–4 central fultoportulae, 6 marginal fultoportulae in 10 μm (Fig. 6G).

####### Ecology.

*Cyclotella choctawhatcheeana* is a small centric diatom from the plankton of water bodies tolerating a wide temperature range. Originally this species was described as a marine species in the northern Gulf Coast of Florida; it is also recorded from several localities in Florida Bay and its global distribution is discussed (Prasad et al. 1990). For Germany, it was described from the River Schlei close to the Baltic Sea (Wendker 1991). Nowadays, it may be classified as an invasive species in brackish waters (Kiss et al. 2012). This species may grow in different seasons and with high and low nutrient availability (Oliva et al. 2008). In turn, some authors note that the existence of this species has a positive linear relationship with nutrient concentration (Jaanus et al. 2009).

####### Distribution.

It was recorded for the first time in Ukraine in our previous investigation near Mykolaiv city of the Southern Bug River and this study confirms its existence in Mykovaiv city and at the downriver sites (Table 1).

*Cyclotella choctawhatcheeana* was recorded as a cosmopolitan species. Its presence has been confirmed in different localities around the world in brackish waters and rivers connected with saline lakes (Prasad et al. 1990). It was found as a component of the phytoplankton in the saline Mexican lake Alchichica (Oliva et al. 2008), in the Baltic Sea, with salinity between 3 and 11‰ (Wendker 1991; Håkansson et al. 1993), and the Salton Sea, with a salinity in excess of 40‰ (Lange and Tiffany 2002). Additionally, it is known from saline lakes in North America and Africa (Carvalho et al. 1995), reservoirs in Russia (Genkal et al. 2020).

###### Cyclotella cryptica

Taxon classificationPlantaeThalassiosiralesStephanodiscaceae

Reimann, J.C. Lewin & Guillard in Phycologia 3: 82, figs 4–11. 1963.

644DEB06-5620-5EB4-87DC-05FC7DCF59F7

####### Morphological description.

**Figure 7.**
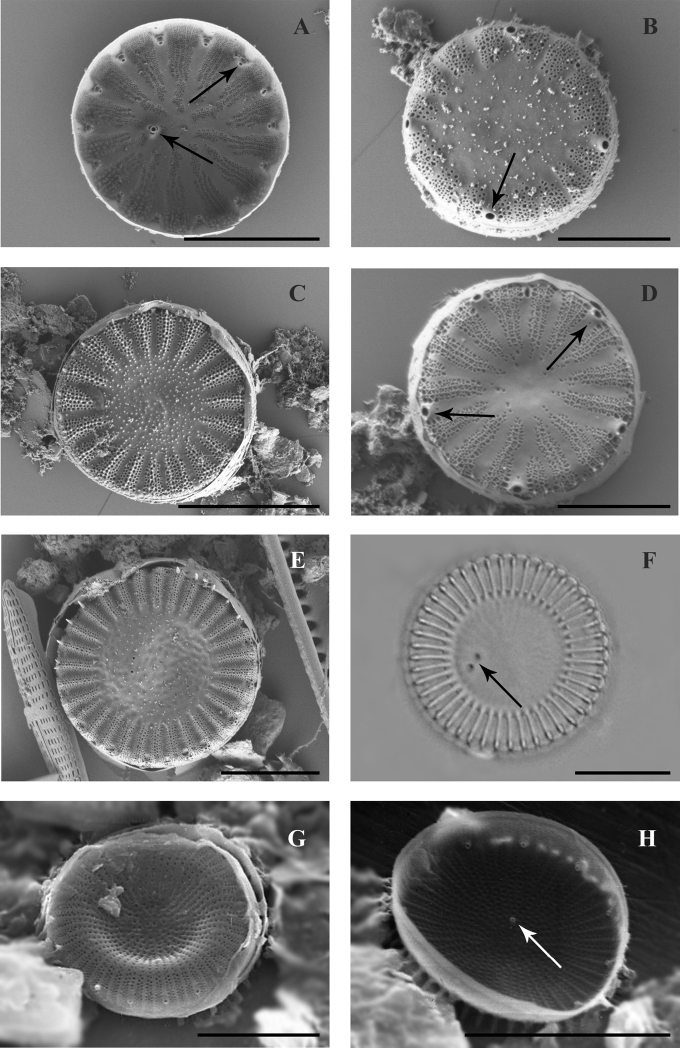
SEM (**A–E, G, H)** and LM images (**F**) of species found in the transitional zone at Mykolaiv city, valve views: **A***Cyclotella cryptica***B***Cyclotella marina***C, D***Cyclotella meduanae***E, F***Cyclotella meneghiniana***G***Cyclostephanos dubius***H***Cyclostephanos invisitatus*, with arrows shown on **A, D, H** marginal and central fultoportulae, **B** marginal fultoportulae, **F** central fultoportulae. Scale bars: 2 μm (**B, D**); 4 μm (**A**); 5 μm (**C, E**); 10 μm (**F–H**).

Frustule cylindrical, the medium part of a valve slightly tangentially undulated, or flat, valve diameter is 6.4–6.7 μm, a clear boundary between edge and central zone is absent, striae wedge-shaped, 8 in 10 μm, single central fultoportula (Figs 6H, 7A).

####### Ecology.

*Cyclotella cryptica* is a planktonic species, known from marine and brackish environments, may be found in high chloride concentrations. It occurs at maximum abundance around 20 °C (Liu and Hellebust 1976; Makarewicz 1987; Mills et al. 1993). *Cyclotella cryptica* is a saprophilic species (Barinova et al. 2019), requires NO_3_ as its source of nitrogen and Ni ions in order to grow autotrophically, however is capable of heterotrophic growth in bottom water or mud enriched in glucose and known to grow mesotrophically (Oliveira and Antia 1984; Saros and Fritz 2000).

####### Distribution.

Valves were identified at the site in the south of Mykolaiv city in the Southern Bug River (Table 1).

It is a widespread species, recorded for Europe, North America, and Asia (Mills et al. 1993; Guiry and Guiry 2021).

###### Cyclotella marina

Taxon classificationPlantaeThalassiosiralesStephanodiscaceae

(Tanimura, Nagumo & M. Kato) Aké-Castillo, Okolodk. & Ector, in Nova Hedwigia, Beih. 141: 267, figs 2–9. 2012.

4ACFEC91-1715-55BD-A267-1C6BA0DA6759

####### Basionym.

Cyclotella atomus var. marina Tanimura, Nagumo & Kato in, Bull. Natn. Sci. Mus., Tokyo, Ser. C. 30: 6–7, figs 3–15. 2004.

####### Morphological description.

Frustule low-cylindrical, valve diameter is 3.2–3.9 μm, a clear boundary between edge and central zone is absent, striae wedge-shaped, 18–20 in 10 μm, central process is absent (Fig. 7B).

####### Ecology.

According to literature data, *C. marina* has a high ecological relevance, with a preference in brackish waters, also inhabiting marine environments even with salinity ranges around 30‰; smaller numbers were recorded for freshwaters under 10‰ (Tanimura et al. 2004; Chung et al. 2010; Aké-Castillo et al. 2012). There are known cases where *C. marina* exists in shallow waters with freshwater discharges. At the same time, the appearance of this taxon is connected to high nutrient concentrations. In addition, cases of blooming of this species are known in the south-eastern Gulf of Mexico (Aké-Castillo et al. 2012).

####### Distribution.

*C. marina* is found in epilithic benthic samples at all investigated sites during this research for the Southern Bug River (Table 1). For Ukrainian territory it is also reported from Khmelnytskyy NPS, (Genkal et al. 2012) and in phytoplankton in Odessa Bay of the Black Sea (Genkal and Terenko 2014).

It is also a common species for Europe, North America, and Asia (Aké-Castillo et al. 2012; Genkal et al. 2012; Guiry and Guiry 2021).

###### Cyclotella meduanae

Taxon classificationPlantaeThalassiosiralesStephanodiscaceae

H. Germ., Fl. Diat. Mass. Armor. 36, pl. 8, fig. 28, pl. 154, figs 4, 4 a. 1981.

63DA2B61-71D1-5D56-87E0-F84A27FE4B60

####### Morphological description.

Frustule low-cylindrical, central part of valve is slightly tangentially undulated or flat, valves 5.8–9.4 μm in diameter, striae wedge-shaped, 6–9 in 10 μm, central process is absent (Fig. 7C, D).

####### Ecology.

*Cyclotella meduanae* was recorded ecologically as a planktonic taxon from different types of water bodies (lakes, reservoirs, rivers), freshwater and brackish, of different halobity, eutraphentic (Kiss et al. 2012; Genkal 2014; Hofmann et al. 2018).

####### Distribution.

This taxon is sporadically found in epilithic benthic samples from the Southern Bug River at two investigated stations (in Mykolaiv City and downstream Mykolaiv) (Table 1). For other Ukrainian waters it has been reported for the Dnipro River (Maystrova et al. 2007), the Danube River (Genkal and Ivanov 1990).

Globally, this taxon is distributed in the Boreal zone, Europe, Asia and North America (Genkal 2014; Genkal et al. 2020).

###### Cyclotella meneghiniana

Taxon classificationPlantaeThalassiosiralesStephanodiscaceae

Kütz., Kieselschal. Bacill.: 50, pl. 30, fig. 68. 1844.

78ABD889-7364-5856-B1F0-09A6FBE38C22

####### Synonyms.

*C. kuetzingiana* Thwaites, *C. rectangula* Bréb. ex Rabenh.

####### Morphological description.

Frustule cylindrical, valves with a tangentially undulated central part, 10.4–33.3 μm in diameter, striae wedge-shaped 5–8 in 10 μm. Central processes (usually from 1 to 9) and spines are present at the mantle of the valve (Fig. 7E, F).

####### Ecology.

*Cyclotella meneghiniana* was recorded as tychoplanktonic, in coastal and estuarine locations with water of varied chemistry (Trigueros and Orive 2000). Its optimal development occurred at temperatures in the range of 20.1–20.6 °C (Stoermer and Ladewski 1976) but it was eurythermal (Gasse 1986). This is a mesopolysaprobic, and eutraphentic taxon, particularly common for shallow, nutrient rich waters, favoured by moderately alkaline conditions (Håkansson 1993; Van Dam et al. 1994).

####### Distribution.

Valves were found at all investigated sites of Southern Bug during this research (Table 1). For Ukrainian territory it has been reported for the Dnipro Estuary, the Southern Bug as well, but near Vinnitsya (300 km upriver form Mykolayiv), the rivers Siverskyi Donets, Dnister, Danube, Dnipro, Desna, Prypiat, Teteriv, Oskol, small rivers in Odessa region and other rivers (Tsarenko et al. 2009).

Concerning global distribution, *Cyclotella meneghiniana* is considered a widespread taxon (Houk et al. 2010); it was also recorded for Berlin, Germany (Geissler and Kies 2003).

#### Order Thalassiosirales Glezer & I.V. Makarova, 1986

**Family Stephanodiscaceae I.V. Makarova in Glezer and Makarova 1986**

##### Genus *Cyclostephanos* Round in Theriot et al. 1987

###### Cyclostephanos dubius

Taxon classificationPlantaeThalassiosiralesStephanodiscaceae

(Hust.) Round in Theriot et al., Brit. Phycol. J. 22 (4): 346. 1987.

234670D8-EEC5-5695-B332-182837CBF9B3

####### Basionym.

*Stephanodiscus dubius* Hust., Krypt.-Fl. Deutschl., 2.Aufl., 7 (1): 367, fig. 192. 1928.

####### Synonyms.

*Cyclotella dubia* Fricke in A.W.F. Schmidt

####### Morphological description.

The frustule is disciform, valve face concentrically undulate, 13.6–21 μm in diameter, striae multiseriate with 9–12 areolae in 10 μm, ribs continue on the curve of a valve, spines grow from each rib (Fig. 7G).

####### Ecology.

*Cyclostephanos dubius* is considered to be a pelagic taxon, planktonic component of both fresh and brackish lakes (Cholnoky 1968). Often indicating meso- to eutrophic conditions, sometimes recorded for hypertrophic lakes (Van Dam et al. 1994; Kirilova et al. 2010). The species is common in flowing and stagnant water in coastal area, oligosaprobic, alkalibiontic, halophilous “0.0–5 g/dm^3^” (Hustedt 1957); pH value above 7.0 (van der Werff and Huls 1957–1974; Foged 1973).

####### Distribution.

This species was found above Mykolaiv city in the Southern Bug River bed (Table 1). From Ukrainian territory, it is known from the Dnipro River, the Dnipro-Bug Estuary (Topachevsky and Oksiyuk 1960; Vladimirova 1971) and the Danube River (Tsarenko et al. 2009).

*Cyclostephanos dubius* is a cosmopolitan species, recorded for Berlin, Germany (Geissler and Kies 2003) and further sites in Europe (Hungary, Dania, Estonia, Moldova, Russia, Ukraine), Asia (Georgia, Russia, Uzbekistan), North America (Canada, USA), Africa (Egypt); Baltic and White Seas (Tsarenko et al. 2009; Kiss et al. 2012; Genkal et al. 2020).

###### Cyclostephanos invisitatus

Taxon classificationPlantaeThalassiosiralesStephanodiscaceae

(M.H. Hohn & Hellerman) Stoermer, E.C. Ther. & Håk. in Theriot, Stoermer and Håkansson, Diatom Res. 2: 256, figs 10 d–f. 1988.

91130038-E4C6-5C96-81F0-A2DF63C16419

####### Basionym.

*Stephanodiscus invisitatus* M.H. Hohn & Hellerman, Trans. Am. Microscop. Soc. 82 (3): 325. 1963.

####### Morphological description.

Frustule disciform, valve face flat, 9.4–14.5 μm in diameter, multiseriate striae 10–14 in 10 μm, ribs are continuing on curve of the valve, spines grow from every rib (Fig. 7H).

####### Ecology.

*Cyclostephanos invisitatus* was recorded as planktonic species from rivers, ponds, lakes, reservoirs and seas, freshwater, brackish and marine waters. Also known from waters of eutraphentic conditions, moderate and higher trophy and moderate alkalinity (Krammer and Lange-Bertalot 2000; Siver et al. 2005; Kirilova et al. 2010; Hofmann et al. 2018).

####### Distribution.

*C. invisitatus* was found 5 km downstream Mykolaiv city (Table 1), and was recorded for this River earlier (Bilous et al. 2012; Bilous et al. 2014; Belous 2016). In turn, it is common for the Dnipro River and there are some findings in the Danube River (Tsarenko et al. 2009).

Probably a cosmopolitan species, known from Europe (Germany, Hungary, Poland, Russia), Asia (Armenia, Azerbaijan, Russia), Northern America (USA), Africa (Egypt); Caspian Sea (Wojtal and Kwandrans 2006; Tsarenko et al. 2009; Kiss et al. 2012; Genkal et al. 2020).

## Discussion

Based on the results above, indicator species for eutrophication in the coastal area of Black Sea waters, which are entering the estuary and then move upstream into the Southern Bug River, were identified. From the investigated taxa the following have been reported as tolerant to nutrient pollution: *Aulacoseira subarctica*, *Cyclotella atomus*, *C. choctawhatcheeana*, *Cyclostephanos dubius*, *Melosira varians*, *Skeletonema subsalsum*, *Stephanodiscus hantzschii*, *S. minutulus* (Hasle and Evensen 1975; Van Dam et al. 1994; Bradbury et al. 2002; Chris et al. 2003; Sarno et al. 2005; Yang et al. 2005; Jaanus et al. 2009; Kirilova et al. 2010; Höglander et al. 2013; Lowe 2015; Reavie and Kireta 2015; Burge and Edlund 2016; Nardelli et al. 2016; Hofmann et al. 2018). Therefore, it is not surprising, that all of them were recorded at Mykolaiv city during different sampling studies and only some of them sporadically at each site.

The conducted research was supplemented by the information concerning the basic biology of centric diatoms, their distribution and occurrence in the transition zone of a freshwater-saline environment. Comparison of the two investigations of 2013 and 2017, revealed the absence of *Melosira varians* in 2017. This might be initialised by changes of ecological conditions near the investigated territory of the Mykolaiv area in the Southern Bug River and eventually by the displacement of this taxon by other representatives of the genus that are better adapted to increasing salinity. The evident displacement representative is *Melosira subglobosa*, which prevailed in the studied area with relatively high abundancies from 57.1 to 80.4% for all centric diatoms over all investigated sites of the transitional zone of the Southern Bug River.

During the sampling research in 2017 a new taxon for Ukraine, *Aulacoseira nivalis*, was found. Since this species is rare, up to now poorly studied and not enough data concerning its ecology and distribution are available, our finding could serve to supplement the existing information. In Hofmann et al. (2018) the taxon is characterised as oligotraphentic and living in dystrophic waters; but this does not seem to correspond to the characteristics of the Ukrainian waters in this area (Mykolaiv regional state administration 2019). Different information for *A. nivalis* is presented in Kulikovskiy et al. (2016), where it is found in alkaline as well as in acidic waters. Ten more taxa found by us in this area are alkalibionts and probably reveal the appropriate conditions for *A. nivalis* in the investigated water body, if all publications are talking about the same taxon. Further focused investigations to establish the appropriate ecology, distribution and identity of *A. nivalis* should be conducted to validate the presented information.

One more interesting finding is Cyclotella atomus var. gracilis that was reported for the first time for the studied area (Tsarenko et al. 2009). For other water bodies in Ukraine, this species is rare and recorded only once for the Dnipro (Maystrova et al. 2007), however, it is probably more widespread in Ukraine, since its varieties Cyclotella atomus var. gracilis and Cyclotella atomus var. atomus are difficult to differentiate using light microscopy, and the nominate one is widespread in Ukraine (Tsarenko et al. 2009). As we have done detailed morphological investigations using LM and SEM microscopy, it gave us the possibility to distinguish C. atomus var. gracilis. This taxon is marked as common for freshwaters, however some authors mention it as an euryhaline species (Kiss et al. 2012). It is supposed that the ecology of this taxon as well as the previous one need further focused investigations.

*Aulacoseira islandica* is also the first confirmation from Swirenko’s investigations of the benthic flora of the lower part of the River in 1925–1926 that has reappeared during our study (Swirenko 1941). As it was mentioned above, this taxon is common for the Dnipro river basin, moreover it is also known for swamps from the eastern part of Ukraine as well as from estuaries in the southern part of the country (Ivanov and Karpeso 1999). Moreover, this is a common species for neighbouring countries in Europe as well as worldwide. *A. islandica* is reported from waters of different trophy levels. Thus, it seems surprising that *A. islandica* was not reported for the Southern Bug river until now, especially for the well explored lower part of the river.

Previously, our investigations revealed ten centric diatom species as new for this territory: seven species (*Aulacoseira subarctica*, *Conticribra weissflogii*, *Cyclostephanos invisitatus*, *Cyclotella atomus*, *C. choctawhatcheeana*, *C. meduanae*, *Thalassiosira faurii*) were found in our study of the sampled material from 2013 (Genkal and Bilous 2015) and three species were discovered in 2017 but considered as rare (*Cyclotella cryptica*, *C. marina*, *Stephanodiscus makarovae*) in our publication from 2019 (Bilous et al. 2019). In this paper, four further species (*Aulacoseira nivalis*, *A. islandica*, Cyclotella atomus var. gracilis, *Melosira subglobosa*, *Skeletonema subsalsum*) were revealed as new to this river. Altogether, for the examined zone of the Southern Bug River 14 centric diatom species were published for the first time for this river due to detailed examination of the species of our 2013 and 2017 sampling studies. The total number of centric diatoms in the studied area shows the existence of 24 centric diatom taxa representing 11 genera (Table 1).

Among the centric diatoms we also found three alien or potentially neophytic species (*Actinocyclus normanii*, *Skeletonema subsalsum*, *Thalassiosira incerta*) that might be considered as an immigration of marine species to freshwaters (Kaštovský et al. 2010; Korneva 2014; Vidaković et al. 2016), consequently their monitoring is important for biodiversity conservation. For Actinocyclus normanii f. subsalsus, here treated as a synonym of the nominate forma, the Ukrainian findings might be comparable to Germany where this taxon migrated from coastal habitats to inland waters (Geissler and Kies 2003; Geissler et al. 2006).

When comparing the composition of species, we found about 5–6 species for each investigated site along the river (Bilous et al. 2012; Bilous et al. 2014; Belous 2016). Our current findings show that the studied sites of the Lower Southern Bug River near Mykolaiv city have centric diatom species numbers a few times higher than the sampling sites analysed in our former studies (Genkal and Bilous 2015). Therefore, we are classifying this area as a transitional zone.

For more specific definitions for transitional zones two ecological terms are currently used (among others), the ecotone and the ecocline. An ecotone, being defined as highly dynamic and usually unstable, results in an environmentally stochastic stress zone. For diatoms an ecotone would mean that each species can be assigned to clear-cut specific zones such as freshwater, brackish, and marine (Attrill and Rundle 2002). The term ecocline in diatoms would refer to an area that due to e.g. physicochemical variation represents a boundary of more gradual, progressive change, meaning that species could more easily migrate from freshwaters through brackish to marine waters and vice versa with less distinct/clear cut zones (Attrill and Rundle 2002). Although this study was not designed to answer this question, the combined data of all sampling researches could indicate that the here studied transitional zone fits the definition of an ecocline.

The occurrence of marine taxa in estuaries and freshwaters below the physiological salt barrier of about 5‰ was discussed in Geissler and Kies (2003) for Hamburg, Germany. The presence of single valves of marine diatoms in eutrophic fresh waters in cities far away from marine habitats was interpreted as air borne valves or part of anthropogenic wastewater impacts (Geissler and Kies 2003). Only taxa with high euryhalinity tolerance are able to move upstream and adapt to eutrophic freshwaters, which have also an increased conductivity.

Although the majority of centric genera are discovered in strictly marine waters, and relatively few of them are present in strictly freshwaters (Kociolek et al. 2015), a growing number of them seem to be able to live in the wide salinity spectrum of brackish waters. This might also be attributed to the increased studies of these large transitional zones. Indeed, in this paper the distribution of centric diatoms near Mykolaiv city according to their ecological characteristics showed the prevailing number of species as estuarine. Mykolaiv city at the lower part of the Southern Bug River represents a river region with salt water inflow into the freshwater river. The salinity here varies in a wide range from 2.39 up to 6.36 g/dm^3^, indicating the effect of marine and freshwater discharge (Governmental portal 2020). Nevertheless, not only salinity but also anthropogenic influences from Mykolaiv city might also add to the higher centric diatom species number at this site. These questions need to be elaborated in detail and in future investigations of this area.

## Conclusion

Due to the presence of many centric diatoms in all types of water bodies, they have often been considered as a cosmopolitan if not ubiquitous group. This makes centric diatoms a very good organism group to be used for bioindication purposes owing to the widescale presence of the environmental conditions suitable for their development within the studied area.

Our study shows that salinity is one of the most influential factors for diatom species composition. The transitional zone of the Mykolaiv area with its changing salinities is offering conditions for the existence of 24 centric diatom taxa representing 11 genera. The occurrence of three marine taxa may be considered as an immigration of marine species into this area due to changing salinities. These trends should be thoroughly monitored for the inland waters of Ukraine in the future. The presented results are a documented contribution to the regional flora of Ukraine.

## Supplementary Material

XML Treatment for Melosira subglobosa

XML Treatment for Melosira varians

XML Treatment for Aulacoseira islandica

XML Treatment for Aulacoseira nivalis

XML Treatment for Aulacoseira subarctica

XML Treatment for Actinocyclus normanii

XML Treatment for Pleurosira laevis

XML Treatment for Conticribra weissflogii

XML Treatment for Thalassiosira incerta

XML Treatment for Thalassiosira faurii

XML Treatment for Minidiscus proschkinae

XML Treatment for Skeletonema subsalsum

XML Treatment for Stephanodiscus hantzschii

XML Treatment for Stephanodiscus makarovae

XML Treatment for Stephanodiscus minutulus

XML Treatment for Cyclotella atomus Hust. var. atomus

XML Treatment for Cyclotella atomus var. gracilis

XML Treatment for Cyclotella choctawhatcheeana

XML Treatment for Cyclotella cryptica

XML Treatment for Cyclotella marina

XML Treatment for Cyclotella meduanae

XML Treatment for Cyclotella meneghiniana

XML Treatment for Cyclostephanos dubius

XML Treatment for Cyclostephanos invisitatus
